# Mesalamine - A Revered Drug for Inflammatory Bowel Disease With Detrimental Effects on the Lung and Heart

**DOI:** 10.7759/cureus.43567

**Published:** 2023-08-16

**Authors:** Ahtshamullah Chaudhry, Jawad Noor, Ghulam Fatima, Saima Batool, Riwad Noor

**Affiliations:** 1 Internal Medicine, Saint Dominic Hospital, Jackson, USA; 2 Internal Medicine, Abbassi Shaheed Hospital, Karachi, PAK; 3 Pathology, Nishtar Medical University, Multan, PAK; 4 Public Health, Nishtar Hospital, Multan, PAK

**Keywords:** lung, cardiomyopathy, inflammatory bowel syndrome, ulcerative colitis (uc), mesalamine

## Abstract

To keep inflammatory bowel disease (IBD) in remission, mesalamine is frequently utilized. It primarily targets the inflammatory response and lowers prostaglandin and leukotriene synthesis. It can be applied topically, orally, or as a suppository. It is typically well tolerated, but occasionally it can cause serious side effects, leading to a variety of medical problems. We describe two cases of severe mesalamine-induced toxicity, one of which manifested as cardiomyopathy and the other as pneumonitis. However, early detection and treatment of the side effects can be lifesaving.

## Introduction

Because of its anti-inflammatory properties, mesalamine is one of the drugs used to treat inflammatory bowel disease (IBD). It is the sulfasalazine's active moiety, which undergoes metabolization to form sulfapyridine and mesalamine. It has no sulfur component but is composed of 5-aminosalicylic acid (5-ASA) without the sulfapyridine carrier molecule [1]. Different types of 5-ASA compounds have been developed to prevent absorption in the proximal gastrointestinal tract. Common mesalamine side effects are headache, diarrhea, bloating, nausea, and hypersensitivity [2]. However, it can also cause serious side effects in the heart and lungs, which were seen in our patients. We hereby describe two patients who developed life-threatening adverse effects from mesalamine affecting the lungs and heart. As demonstrated by our examples, early detection of these side effects and a proper approach can save lives.

## Case presentation

Pulmonary case

A 28-year-old Indian woman with a history of ulcerative colitis (UC) presented to the hospital with a two-week history of fever and cough. She described her fever as intermittent, with two to three episodes per day, associated with chills, and with the highest temperature reading of 102.9 °F. Her cough was dry and had no relation to a fever. The medication list included only mesalamine 800 mg times a day and ibuprofen as needed. Four weeks before presenting to the hospital, she was diagnosed with UC in India and started on mesalamine, which she has been on for more than three weeks. 

On review of systems, there was no hemoptysis, sick exposure, change in appetite, night sweats, orthopnea, weight loss, joint pain, or skin rash. On the day of admission, her temperature was 99.8 °F, her pulse was 111 beats per minute, blood pressure was 97/53 mm Hg, and there was decreased bilateral air entry on auscultation of her lungs. She appeared to be comfortable, and the rest of the examination was unremarkable. The blood workup was normal for electrolytes, liver, and renal functions. On a complete blood count (CBC), she had normocytic anemia (hemoglobin: 9.9 mg/dl, mean corpuscular volume: 88), 0 eosinophils on differential, a C-reactive protein of 6.6 mg/dl, and an erythrocyte sedimentation rate of >140.

**Figure 1:**
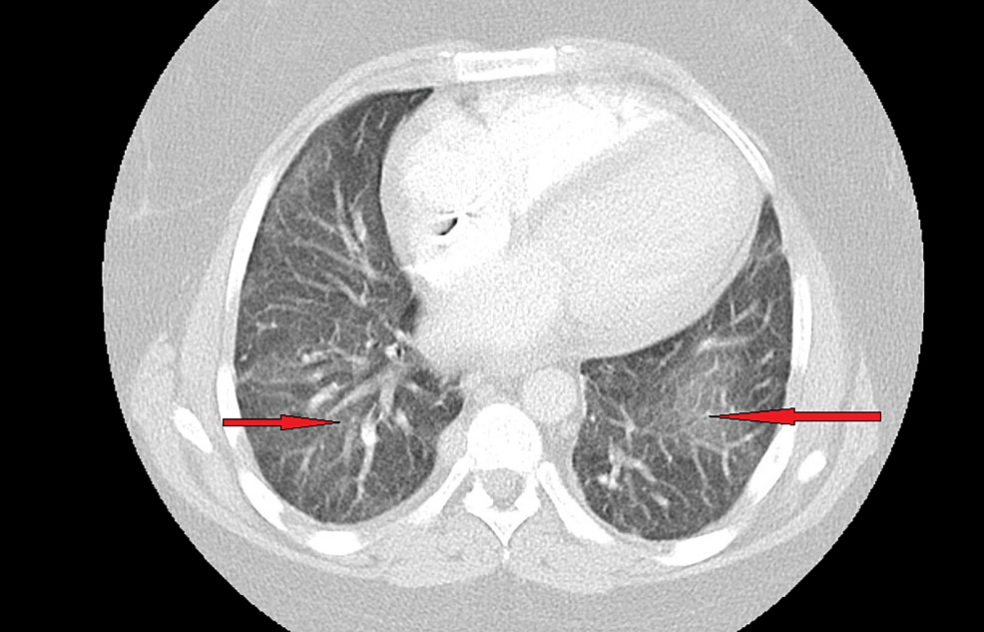
CT angiogram of chest showing patchy ground glass opacities at bilateral lung bases

A CT angiogram of the chest was done, and it showed no pulmonary embolism, but it showed patchy grand glass opacities at bilateral lung bases (Figure 1). Tuberculosis was ruled out with three negative sputum acid-fast bacilli. Blood work came back negative for the human immunodeficiency virus, rheumatoid factor, antinuclear antibody, and complements.

She continued to spike her fever despite being on broad-spectrum antibiotics for three days. Blood culture showed no organisms. The patient underwent a bronchoscopy, and the bronchial culture was negative. Bronchial lavage showed many pigmented macrophages, lymphocytes, and a few neutrophils. At this time, a potential connection between UC and pneumonitis was considered, although she had no gastrointestinal manifestations. Her mesalamine was stopped, and after one day of being off mesalamine, there were no further episodes of fever. Therefore, stopping mesalamine and improving symptoms raised clinical suspicion of mesalamine-induced interstitial pneumonitis. Antibiotics were stopped, and she was started on methylprednisolone 60 mg (1 mg/kg) IV three times a day. After being on steroids for two to three days, she started to feel better, energetic and reported being back to her baseline. She was discharged home on a Medrol 4 mg dose pack for 21 days and was advised to follow up with a gastroenterologist for further UC management.

Cardiology case

A 32-year-old Caucasian male with a past medical history of Crohn's disease was admitted to the hospital for palpitations and shortness of breath (SOB). Two months before the admission, he was diagnosed with Crohn's disease and was started on infliximab 400 mg (5mg/kg) (weeks zero, two, and six), which was completed. After that, he was placed on mesalamine 1 gram three times a day. He started experiencing palpitation and SOB after taking mesalamine for one week. He was ambulatory and had no complaints before the medication was started. His effort tolerance was New York Heart Association (NYHA) III. There is no family history of heart condition. 

**Figure 2:**
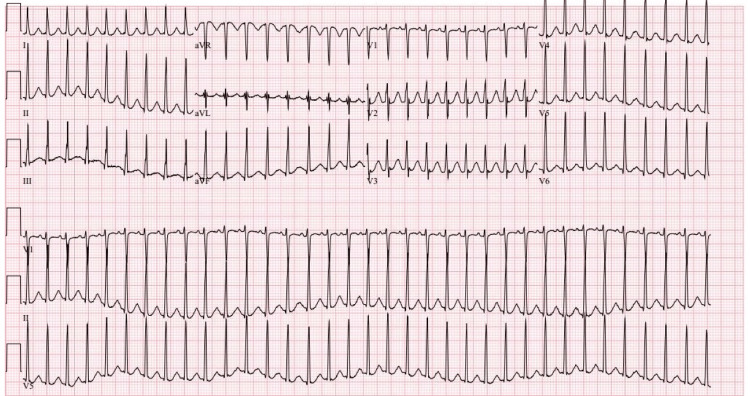
Narrow complex tachycardia

On the day of the presentation, his electrocardiogram (EKG) showed regular narrow complex tachycardia (Figure 2). A cardiovascular examination was significant for tachycardia with normal heart sound intensity, and a gallop was appreciated; venous pressure was normal. His lungs were clear on auscultation, his abdomen was soft and non-distended, and there was no hepatosplenomegaly. Pulmonary embolism was ruled out with chest CT-angiogram, and it revealed normal lung parenchyma, pericardium, and no central pulmonary artery filling defect. 

Blood work was unremarkable except for hemoglobin of 10.3 mg/dl, albumin of 2.7 g/dl, troponin of 0.06 ng/ml, urine drug screen was negative, the thyroid-stimulating hormone of 0.86 UIU/ml, eosinophils mild elevated (9%), proBNP N Terminal was 682 pg/ml with normal kidney functions. Echocardiogram showed moderate global left ventricular systolic dysfunction with an estimated ejection fraction of 35%.

Left heart catheterization was done and it showed normal coronary arteries. A presumptive diagnosis of mesalamine-induced cardiomyopathy was made, and the medication was stopped. His SOB and palpitation started to improve. Pt started to improve clinically, and he was discharged home. He was seen at the cardiologist clinic for a follow-up appointment after two weeks, and he reported being back to his baseline and relief of palpitations and shortness of breath. Pt symptoms started after a few days of being started on mesalamine and improved after stopping the drug, which argues against infliximab causing cardiomyopathy.

## Discussion

IBD is a state of altered immune regulation leading to a prolonged mucosal inflammatory response by increased levels of interleukin-1, 6, and 8, tumor necrosis factor (TNF) alpha, and CD4+ [3]. The presence of luminal bacteria and the absence of regulatory proteins in the mucosal immune system are generally required for the development of intestinal inflammation [4]. Mesalamine formulations allow for earlier release in the more proximally area of the small intestine [5]. The proposed mechanism by which amino salicylates work is by inhibiting cyclooxygenase and lipoxygenase pathways to decrease the synthesis of prostaglandins and leukotrienes. mesalamine can also inhibit the effects of TNF-alpha, which normally releases cytokines that cause inflammation [4]. Other processes inhibited are the platelet-activating factor and the production of oxygen radicals. Mesalamine also acts by decreasing the production of prostaglandins, leukotriene B4 and certain hydroxy fatty acids [6].

Pulmonary effects caused by mesalamine are rare and have a limited understanding of the pathogenesis. However, two mechanisms may be responsible for these side effects, which include a dose-dependent toxicity mechanism and secondly, an immunologic mechanism that is dose-independent. Both mechanisms lead to similar symptoms exhibited by the patients, which include fatigue, non-productive cough, fever, dyspnea, and chest pain [7]. These symptoms usually appear after one to six months of treatment with mesalamine, but in some cases, it occurs after just a few days. CT scan of the chest would demonstrate pulmonary nodules and an interstitial infiltrate with ground glass, persistent with our findings in our patient. Further testing shows some significant findings, such as bronchoalveolar lavage, which shows elevated eosinophils or lymphocytes with a reduction of the CD4/CD8 ratio. Pulmonary function testing demonstrates a reduced diffusing capacity of the lungs for carbon monoxide (DLCO). Lastly, a histological slide would demonstrate interstitial lymphocytic infiltrates, alveolar eosinophilic infiltrates, alveolar fibrosis, and non-necrotizing granulomas [7].

Distinguishing the difference between mesalamine-induced or IBD-related pulmonary manifestation can be difficult. The areas of the lungs can help further distinguish the cause of the pulmonary symptoms. Mesalamine-induced lung disease usually affects the lung parenchyma, whereas IBD-related pulmonary manifestations typically involve the upper respiratory tract [7]. The use of further testing, such as bronchoalveolar lavage, would help with determining the diagnosis of mesalamine-induced lung disease. Treatments for mesalamine-induced lung toxicity include decreasing the dosage. However, if the symptoms are severe, then stopping mesalamine is advised. If further respiratory symptoms continue, glucocorticoid therapy with prednisone (1 mg/kg per day) should be considered [7].

Cardiac manifestations from the extraintestinal manifestation of IBD are a very rare complication that occurs as pericarditis in most cases. Normally it presents many years following the initial diagnosis. More often, cardiac symptoms in these patients are usually due to a drug-induced cause, such as an idiosyncratic hypersensitivity reaction and a drug-induced lupus-like syndrome [7]. Mesalamine-induced cardiac toxicity is not a life-threatening manifestation. Cardiotoxicity secondary to mesalamine may present two to four weeks after the drug has been initially started. Symptoms include lassitude or fatigue with a pericardial friction rub found on physical examination. Screening tests, such as the Naranjo algorithm scale, are used to determine the cause of the patient's cardiac symptoms and if it is caused by an adverse drug reaction. A number >9 suggests a definite cause of cardiac symptoms was due to a drug. Other supporting factors, such as increased eosinophilia, would help further support mesalamine-induced cardiac toxicity. Electrocardiography done on these patients would show evidence of myocarditis with nonspecific findings of flipped T waves, ST wave depression, or possible ST wave elevation, the latter being more common [8]. Cardiac echocardiography commonly showed the presence of pericardial effusion, consistent with pericarditis, which could lead to a cardiac tamponade if it is acutely accumulated [8].

Laboratory findings from mesalamine-induced cardiac toxicity can show leukocytosis with prominent eosinophilia. Evidence of inflammation was seen by elevation of the erythrocyte sedimentation rate and C-reactive protein. N-terminal pro-brain natriuretic peptide and troponin were also shown to be elevated [8]. Onset is key in distinguishing if the cause of the patient's cardiac symptoms is, in fact, due to mesalamine. Given the signs and symptoms occurring within a short period after initiation of the mesalamine therapy, this would support the idea of a drug-induced injury. Most of the patients presented within 28 days of starting mesalamine [8].

Treatment for these symptoms includes mesalamine suspension and administration of non-steroidal anti-inflammatory drugs or corticosteroids [8]. Treatment of cardiomyopathy associated with IBD requires adequate disease control.

## Conclusions

IBD is a complicated condition that calls for complicated management. Due to its propensity for extra-intestinal manifestation and multisystem involvement, it commonly presents as a clinical puzzle. When mesalamine is used to treat IBD, it is important to monitor any new symptoms, such as dyspnea and chest pain. We described two cases of severe mesalamine-induced toxicity where the cardiac symptoms were diagnosed as cardiomyopathy, and the pulmonary symptoms were characterized as pneumonitis. Although uncommon, these side effects are diagnosed after ruling out all other causes, as was done with our patients. A hint of mesalamine toxicity can also be seen in the timeline leading up to the development of these symptoms. Early recognition and treatment of these severe adverse effects can be lifesaving. 
